# Carcass and body organ characteristics of broilers supplemented with dietary sodium and sodium salts under a phase feeding system

**DOI:** 10.1186/2055-0391-56-4

**Published:** 2014-05-15

**Authors:** Mirza Muhammad Haroon Mushtaq, Rana Parvin, Jihyuk Kim

**Affiliations:** Poultry Science Division, National Institute of Animal Science, RDA, 114, Sinbang 1-gil, Seonghwan-eup, Seobuk-gu, Cheonan-si, Chungcheongnam-do 331–801 Republic of Korea

**Keywords:** Sodium, Salt, Carcass and body organ characteristics, Phase feeding program, Broiler

## Abstract

The effect of sodium and sodium salts on carcass and body organ characteristics of broilers under a four phase feeding program were investigated. A basal diet (0.08% dNa with NaCl) was formulated and one of two sources of dNa (NaHCO_3_ and Na_2_SO_4_) were supplemented to obtain four different percentages of dNa (0.17, 0.26, 0.35, and 0.44%) for each treatment. There was a linear decrease in dressing percentage (DP) with source × level interaction (p ≤ 0.001), while there was a linear increase in breast yield and thigh yield with increasing dNa supplementation (p ≤ 0.001). Chicks fed 0.35% NaHCO_3_ and 0.44% dNa Na_2_SO_4_ supplemental salts had lower abdominal fat (p ≤ 0.04). Chicks that received increasing levels of dNa (from 0.17 to 0.44%) showed increasing gizzard weight (p ≤ 0.002) and decreasing spleen weight (p ≤ 0.02). When both salts were supplemented at 0.26% dNa, the chicks showed their lowest bursa weight (p ≤ 0.001). Consequently, chicks at higher dNa showed an increase in breast and thigh meat yield, and increasing capacity of their digestive organ. The higher levels of dNa should be tested with other cations and anions to fully understand acid base homoeostasis.

## Introduction

Sodium (Na^+^), the principal cation of extracellular fluid, is involved in numerous functions including the regulation of extracellular fluid volume, acid base balance, cell membrane potential, nerve function, and the absorption of glucose and amino acids ([1]; Leeson and Summers, [2]). Dietary Na (dNa) and chloride (Cl) are inexpensive in terms of meeting dietary requirements, as Pakistan has huge reserves of sodium chloride (NaCl) [3].

Mongin [1] described the effect and interrelationship of Na^+^, K^+^, and Cl^−^ in an equation for dietary electrolyte balance (DEB = Na + K-Cl, mEq/kg diet). Rondon et al*.*[4] and Borges et al*.*[5] reported different DEB values with different concentrations of Na^+^, K^+^, or Cl^−^. Recently, Mushtaq et al*.*[6, 7] tested 3 levels of dNa and dCl adjusted with dK and reported that different ions acted differently to yield a similar DEB of 250 mEq/kg. The addition of various salts changes osmotic balance by contributing electrolytes [5, 8–10]. Most researchers consider Na-bicarbonate (NaHCO_3_) as the best supplemental salt for Na^+^ and HCO_3_^−^[11–16]. Sodium sulphate (Na_2_SO_4_) induced severe blood acidosis when it was compared with other sulphate sources in growth trials; hence it was suggested that the acidic properties of sulphates are directly linked to supplemental salts [16, 17].

A significant effect of temperature on abdominal fat was found for treatments (5% KCl or 5% NaHCO_3_ in water) under normal but not under cyclic environmental conditions [18]. Sharma and Gangwar [8] reported a decrease in the concentration of Na^+^ and K^+^ in the breast and thigh muscles of broilers from 4 to 8 wks old under high temperature (32°C). The author, moreover, observed that breast muscles had significantly lower Na^+^ and higher K^+^ concentrations than thigh muscles. Pourreza and Edriss [19] reared straight-run broilers at 20°C or 30°C and noticed that the high temperature decreased slaughter, carcass, and abdominal fat weight and increased dressing percentage. Johnson and Karunajeewa [11], Karunajeewa et al. [20], and Borges et al. [21] observed no effect on carcass and abdominal fat yield under different DEB treatments under normal environmental condition.

The development of housing systems and genetics has necessitated a look into the changing nutrient requirements of current broilers. The present study evaluated the effect of dietary sodium supplementation with the applicability of DEB using different sodium salts on carcass and body organ characteristics of a modern broiler strain fed under a four phase feeding program.

## Materials and methods

All experimental birds and procedures were maintained in compliance with laws proposed by the Advanced Studies and Research Board, University of Veterinary and Animal Sciences, Lahore, Pakistan.

### Birds’ husbandry

A total of 1280 day-old straight-run Hubbard broiler chicks (Hubbard × Hubbard) were given one of eight dietary treatments replicated four times; under this system each replicate contained 40 birds (without considering the sex). Each replicate pen was equipped with a separate overhead, transparent, and volume-graduated 20 L water bottle linked to a nipple drinker line. Water bottles were cleaned and filled with fresh water on a daily basis. One flat bottom round feeder was provided for each experimental pen. Birds were housed in environment control systems where variation in temperature and relative humidity were recorded and maintained according to the production manual [22]. Continuous light was provided 24 h for the first 3 days and then a 23 L:1D light pattern was adopted for the rest of the experimental period. Fresh sawdust (7.5 cm deep) was used as litter material over a concrete floor. For the first 3 days, the house temperature was maintained at 32°C and thereafter reduced by 0.5°C per day until 24°C was attained at d 19.

Birds were vaccinated against Newcastle Disease (ND) plus Infectious Bronchitis viruses at 4 d, Infectious Bursal Disease virus at 8 d and again at 14 d; Hydropericardium Syndrome virus at 18 d, and ND-Lasota strain at d 22.

### Dietary plan and experimental design

A basal diet with dNa, K and Cl at 0.08, 0.71, and 0.20%, respectively, with a DEB value of 160 mEq/kg (Table 1 and Table 2) was formulated. For this purpose, a large batch of basal diet was prepared for each phase and then experimental diets were prepared such that four levels of dNa (0.17, 0.26, 0.35, and 0.44%) were supplemented to this basal diet with either commercially available feed-grade sodium bicarbonate (NaHCO_3_) or sodium sulphate (Na_2_SO_4_). The levels of dNa corresponded to DEB values of 200, 240, 280, and 320 mEq/kg, respectively. The experimental period was divided into four phases: pre-starter (1 to 10 d), starter (11 to 20 d), grower (21 to 33 d), and finisher (34 to 42 d) which met or exceeded the nutrient specifications recommended by the Hubbard management guide ([22]; Table 2) except for amino acid composition.

**Table 1:** **Ingredient composition of basal diets fed four levels of sodium with two sources of sodium salts at different stages of growth in broilers** ^**1)**^

Ingredients (%)	Pre-starter	Starter	Grower	Finisher
	(1 – 10 d)	(11 – 20 d)	(21 – 33 d)	(34 – 42 d)
Corn	46.93	47.54	67.46	67.94
Broken rice	13.46	16.16	0.07	−
Soybean meal	27.63	29.11	26.93	24.47
Canola Meal	6.39	1.24	−	−
Oil^2)^	1.62	2.21	1.88	3.94
DCP	2.16	2.02	1.89	1.60
Limestone	1.04	0.94	1.05	1.10
L-Lysine HCl	0.23	0.22	0.20	−
L-Lysine sulphate	−	−	−	0.34
NaCl	0.15	0.16	0.16	0.16
KCl	0.04	0.03	0.05	0.14
DL-methionine	0.20	0.20	0.17	0.17
L-threonine	0.05	0.07	0.03	0.04
Premix^3)^	0.10	0.10	0.10	0.10

^1)^All diets were supplemented with four levels of either NaHCO_3_ (0.33, 0.66, 0.99, or 1.32%) or Na_2_SO_4_ (0.28, 0.56, 0.84, or 1.12%) to make final Na concentrations of 0.17, 0.26, 0.35, or 0.44%, respectively. The basal diet contained 0.08% Na.

^2)^Residual bakery oil.

^3)^Provides per kg of finished diet: vitamin A, 12 mg; vitamin D_3_, 7 mg; vitamin E, 100 mg; vitamin K_3_ (50% as MNB), 3 mg; vitamin B_1_ (98%), 3 mg; vitamin B_2_ (800,000 mg), 12 mg; vitamin B_3_ (niacin; 99%), 600 mg; vitamin B_6_ (98%), 4 mg; vitamin B_9_ (folic acid; 95%), 2 mg; vitamin B_12_ (0.10%), 20 mg; biotin (0.10%), 5 mg; Ca-Pentothenate (98%), 12 mg; cholin (70% as choline sodium), 1 g; MnO (60%), 169 mg; FeSO_4_ (21%), 200 mg; ZnSO_4_ (36%), 150 mg; CuSO_4_ (25%), 40 mg; Se (sodium selenite 0.40%), 100 mg; KI (68%), 2 mg; salinomycin, 60 mg; zinc bacitracin (as Albac 10%), 50 mg.

**Table 2:** **Nutrient composition of basal diets for different phases of birds fed four levels of sodium with two sources of sodium salts** ^**1)**^

Nutrients	Pre-starter	Starter	Grower	Finisher
	(1 – 10 d)	(11 – 20 d)	(21 – 33 d)	(34 – 42 d)
ME (kcal/kg)	2900	3000	3000	3147
Crude Protein (%)	21.00	20.00	19.00	18.00
Calcium (%)	1.00	0.90	0.90	0.85
Available Phos. (%)	0.45	0.42	0.40	0.35
Sodium (%)	0.08	0.08	0.08	0.08
Potassium (%)	0.71	0.71	0.71	0.71
Chloride (%)	0.20	0.20	0.20	0.20
DEB^2)^ (mEq/kg)	160	160	160	160
Dig Lys (%)	1.10	1.05	0.97	0.93
Dig Met/Dig Lys	0.45	0.45	0.44	0.44
Dig Met + Dig Cys/Dig Lys (%)	0.72	0.71	0.72	0.72
Dig Thr/Dig Lys	0.66	0.67	0.66	0.66
Dig Try/Dig Lys	0.18	0.18	0.18	0.17

^1)^NaHCO_3_ and Na_2_SO_4._

^2)^Dietary Electrolyte Balance (mEq/kg) = (% Na × 10,000 / 23) + (% K × 10,000 / 39.1) – (% Cl × 10,000 / 35.5).

All ingredients were assayed for their proximate composition [23] prior to diet formulation and actual values were used in the formulation. The Na^+^ and K^+^ contents of each diet were analyzed by flame photometer [23] and Cl^−^ content was analyzed by titration with AgNO_3_^−^[24]. Prior to starting the experiment, the Na^+^, K^+^, and Cl^−^ contents of the final diet were verified. The ME of each ingredient was calculated by the appropriate regression equation suggested by the NRC [25]. The amino acid composition of each ingredient was calculated using AminoDat™ 3.0 Platinum (Degussa AG, Germany) based on the DM and CP contents of each ingredient [6]. The amino acid composition of each diet met or exceeded the ideal amino acid ratio suggested by Han and Baker [26]. The experiment, offering mash diets, lasted until 42 d of age.

### Growth response

Feed intake (FI; g/bird), BW gain (BWG; g/bird) and feed-to-gain ratio (FG; g:g) were recorded at the end of the experiment. The feed was withheld for 6 h before weighing the birds to ensure the emptying of the digestive tract of the bird. Mortality was recorded on a daily basis and dead bird was weighed prior to removal to correct FG.

### Carcass and organ characteristics

At the end of 42 d, two birds were randomly selected from each replicate and subjected to carcass and body organ evaluation. The feed was withheld for 6 h before slaughter to ensure emptying of the digestive tract. Carcass and body organ responses were evaluated in terms of dressing percentage, breast, thigh, abdominal fat, gizzard, proventriculus, heart, liver, kidney, spleen, pancreas, bursa, gallbladder, intestine, and lung weights, and for the shank and intestine lengths (Table 3 and Table 4). The carcass and body organ weights were taken on a fresh basis. Dressing percentage (DP) was calculated by dividing dressed weight, without viscera, by live weight and multiplied by 100. The weight of the abdominal fat pad was expressed as DP (without visceral weight), while the weights of other body organs (gizzard, proventriculus, heart, liver, kidney, spleen, pancreas, bursa, gall bladder, lungs, and intestine) were taken as a percentage of dressing weight (with visceral weight). Intestinal length was measured (in centimeters) from the start of the duodenal loop to the ileocaecal junction [27].

**Table 3:** **Effect of dietary sodium and sodium salts on growth performance of broilers on day**

Item	BW gain (g/bird)	Feed intake (g/bird)	Feed:gain (g:g)
Dietary Na (%)			
0.17	1,893	3,564	1.95
0.26	1,959	3,634	1.80
0.35	1,883	3,605	1.89
0.44	1,864	3,622	1.95
*SEM*	*30.5*	*45.9*	*0.076*
Salts			
NaHCO_3_	1,884	3,604	1.88
Na_2_SO_4_	1,916	3,608	1.91
*SEM*	*21.6*	*32.4*	*0.053*
Na × Salts			
0.17 × NaHCO_3_	1,941	3,586	1.82
0.26 × NaHCO_3_	1,941	3,631	1.83
0.35 × NaHCO_3_	1,829	3,649	1.91
0.44 × NaHCO_3_	1,824	3,551	1.98
0.17 × Na_2_SO_4_	1,844	3,542	2.08
0.26 × Na_2_SO_4_	1,977	3,637	1.76
0.35 × Na_2_SO_4_	1,936	3,560	1.88
0.44 × Na_2_SO_4_	1,905	3,694	1.91
*SEM*	*43.1*	*64.9*	*0.107*
ANOVA	*———————— Probability ————————*		
Na	NS	NS	NS
Na_L_	NS	NS	NS
Na_Q_	NS	NS	NS
Salt	NS	NS	NS
Salt × Na	0.036	NS	NS

NS - Non-significant.

Na_L_ and Na_Q_ are linear and quadratic terms for Na, respectively.

**Table 4:** **Effect of dietary sodium and sodium salts on carcass and body organ responses of broilers**

Item	Dressing weight^1)^	Breast weight^2)^	Thigh weight^2)^	Intestinal weight^3)^	Abdominal fat^1)^
Dietary Na (%)					
0.17	56.12	31.85	45.26	58.2	3.01
0.26	54.18	32.98	46.28	54.3	3.12
0.35	52.94	33.79	47.34	54.9	2.44
0.44	52.07	34.31	48.30	56.1	2.61
*SEM*	*0.423*	*0.347*	*0.627*	*1.15*	*0.24*
Salts					
NaHCO_3_	53.95	33.16	46.28	56.3	2.74
Na_2_SO_4_	53.70	33.30	47.31	55.4	2.84
*SEM*	*0.299*	*0.246*	*0.443*	*0.81*	*0.17*
Na × Salts					
0.17 × NaHCO_3_	55.68	32.11	45.29	58.8	2.96
0.26 × NaHCO_3_	54.85	32.56	45.05	56.2	2.93
0.35 × NaHCO_3_	53.38	33.57	46.79	58.7	1.93
0.44 × NaHCO_3_	51.90	34.40	48.01	51.6	3.12
0.17 × Na_2_SO_4_	56.56	31.60	45.24	57.7	3.06
0.26 × Na_2_SO_4_	53.50	33.40	47.51	52.4	3.31
0.35 × Na_2_SO_4_	52.51	34.01	47.90	51.1	2.94
0.44 × Na_2_SO_4_	52.24	34.22	48.58	60.7	2.11
*SEM*	*0.599*	*0.492*	*0.886*	*1.62*	*0.35*
ANOVA	*———————— Probability ————————*				
Na	≤0.001	≤0.001	0.008	NS	NS
Na_L_	≤0.001	≤0.001	≤0.001	NS	NS
Na_Q_	NS	NS	NS	0.03	NS
Salt	NS	NS	NS	NS	NS
Salt × Na	NS	NS	NS	≤0.001	0.04

NS - Non-significant.

^1)^% of live weight (without visceral organs).

^2)^% of dressed weight (with organs weights).

^3)^measured in grams.

Na_L_ and Na_Q_ are linear and quadratic terms for Na, respectively.

### Water characteristics

Water alters carcass responses because of its concentrations of electrolytes (Na^+^, K^+^ and Cl^−^); therefore, supplied water was analyzed for these electrolytes. Water characteristics were also recorded twice (morning and noon) daily to check pH by pH meter (LT-Lutron pH-207 Taiwan) and dissolved oxygen by DO meter (DO; YSI 55 Incorporated, Yellow Springs, Ohio, 4387, USA). Moreover, temperature, electrical conductivity (EC), total dissolved solids (TDS), and salinity were recorded by the Combo meter (H M Digital, Inc. CA 90230; Table 5). These observations were randomly recorded from different replicates.

**Table 5:** **Drinking water properties during the experimental period**

Phase	Item	Salinity	TDS^1)^	EC^2)^	Temperature	pH	DO^3)^
Phase 1	Max	1.30	1284	1.39	29.7	7.49	5.40
	Min	1.20	1198	1.27	26.2	7.31	3.90
	Average	1.25	1251	1.32	27.3	7.40	4.65
Phase 2	Max	1.20	1111	1.21	26.4	7.35	5.07
	Min	1.20	1060	1.06	25.8	7.17	3.70
	Average	1.20	1088	1.12	26.0	7.27	4.39
Phase 3	Max	1.30	1187	1.24	25.2	7.40	5.60
	Min	1.20	1108	1.08	24.3	7.21	3.70
	Average	1.22	1144	1.15	24.9	7.33	4.65
Phase 4	Max	1.20	1194	1.21	24.8	7.33	5.07
	Min	1.20	1110	1.07	24.0	7.23	3.70
	Average	1.20	1141	1.11	24.2	7.29	4.39

^1)^TDS - Total Dissolved Solids.

^2)^EC - Electric Conductivity.

^3)^DO - Dissolved Oxygen.

Salinity, TDS, EC, temperature, and DO were measured as parts per thousand (ppt), parts per million (PPM), millisemen/centimeter (mS/cm), centigrade (°C) and milligram/litre (mg/L), respectively.

### Statistical analyses

The experiment was executed under a completely randomized design with factorial arrangement using four levels of dNa from two salt sources. The experimental pen was an experimental unit. The data obtained at the end of the experiment were subjected to ANOVA using GLM of Minitab 15.1 (Minitab Inc., State College PA). A statistical significance of 0.05 was used unless stated otherwise.

## Results and discussion

Water was evaluated on a daily basis for quality parameters including temperature, pH, EC, TDS, DO, and salinity (Table 5). At the start of the experiment, water was also analyzed for its sodium absorption ratio (25.6) and residual sodium carbonate (9.02). As the concentration of various minerals (cations plus anions) and values of other quality parameters in drinking water could alter the electrolyte concentration of digesta ([12, 28]; b) their concentration in water was evaluated. The water electrolyte concentration was too low to impact carcass and organ yields. Water pH values (7.17–7.49) were within the range (6.0–8.5) considered optimal for broiler performance [28–30]. Previous reports [31, 32] showed retarded growth up to a pH level of 6.3. The water TDS level ranged from 1000–3000 ppm was considered satisfactory for broilers by Chiba [33], however the analyzed values i.e. 1060-1284 did not appear to disturb the present experiment.

All growth responses were unaffected by dietary treatments, except for sodium level and salt interaction on BW gain (BWG; p ≤ 0.036; Table 3). The supplementation of Na_2_SO_4_ at 0.26% showed higher BWG at day 42. However this change in BWG was not sufficient to positively influence feed:gain (FG).

Carcass and intestinal responses were affected by supplementation of dNa from NaHCO_3_ and Na_2_SO_4_ (Table 4). A highly linear drop in DP (p ≤ 0.001) was observed with increasing supplementation of dNa. Mushtaq et al. [7] observed no difference in DP with increasing dNa from 0.20 to 0.30%. Their difference in results might be due to the lower levels of dNa studied. Salt sources or interaction effects in the present study were not beneficial on DP *per se.* Breast (p ≤ 0.001) and thigh (p ≤ 0.001) meat yield increased with increasing supplementation of dNa (Table 4). This contradiction in results might be due the measurement of breast and thigh meat as a percent of dressing weight with organs. These findings were not in line with the results of Mushtaq et al. [7] who observed reduced breast and leg meat by increasing dNa from 0.20 to 0.30%. This difference could be due to heat stress conditions in their experiment as more nutrients, consumed to maintain acid base balance, may not be converted to meat.

The abdominal fat pad was affected with source × level interaction (Table 6). The abdominal fat pad was lowest at 0.35% dNa in diets supplemented with NaHCO_3_. A similar response of abdominal fat to a high level of dNa (0.30%) was observed by Mushtaq et al. [7]. In the present study, increasing dNa under normal physiological conditions did not disturb basal metabolism and energy was mainly utilized for meat production and not wasted as abdominal fat.

**Table 6:** **Effect of dietary sodium and sodium salts on body organ weights of broilers at the end of the experiment**

Item	Gizzard	Kidney	Spleen	Bursa
	**—————** % of dressed weight^1)^ **—————**			
Dietary Na (%)				
0.17	2.41	0.31	0.13	0.23
0.26	2.57	0.36	0.08	0.15
0.35	2.98	0.37	0.07	0.21
0.44	2.99	0.37	0.08	0.25
*SEM*	*0.15*	*0.02*	*0.02*	*0.02*
Salts				
NaHCO_3_	2.85	0.42	0.09	0.21
Na_2_SO_4_	2.62	0.28	0.09	0.22
*SEM*	*0.10*	*0.02*	*0.01*	*0.01*
Na × Salts				
0.17 × NaHCO_3_	2.72	0.42	0.11	0.16
0.26 × NaHCO_3_	2.65	0.47	0.10	0.18
0.35 × NaHCO_3_	2.98	0.41	0.08	0.22
0.44 × NaHCO_3_	3.05	0.39	0.07	0.26
0.17 × Na_2_SO_4_	2.11	0.21	0.15	0.29
0.26 × Na_2_SO_4_	2.48	0.24	0.61	0.13
0.35 × Na_2_SO_4_	2.98	0.33	0.06	0.20
0.44 × Na_2_SO_4_	2.93	0.35	0.08	0.24
*SEM*	*0.21*	*0.04*	*0.02*	*0.02*
ANOVA	*———————— Probability ————————*			
Na	0.01	NS	NS	0.001
Na_L_	0.002	NS	0.02	NS
Na_Q_	NS	NS	NS	0.01
Salt	NS	≤0.001	NS	NS
Salt × Na	NS	0.03	NS	0.001

NS - Non-significant.

^1)^% of dressed weight (with organ weights).

Na_L_ and Na_Q_ are linear and quadratic terms for Na, respectively.

The interaction (level × source) effect was found to change intestinal weight (p ≤ 0.001); the lowest weight (51.6 vs. 51.1 gm) was recorded at 0.44% (NaHCO_3_) and 0.35% dNa (Na_2_SO_4_). Intestinal weight reflects the gut’s capacity to absorb nutrients, which reflects better health; therefore, higher levels of dNa showed a lower carcass yield.

Organ weights of the proventriculus, heart, liver, pancreas, gall bladder, lungs, and intestinal and shank lengths were measured and found to be non-significant (data not shown). In contrast, weights (% of dressed weight) of gizzard, kidney, spleen and bursa significantly changed (Table 4). Gizzard weight was increased linearly with increasing levels of dNa from 0.17 to 0.44% (P ≤ 0.002; main effect). The increased weight of the gizzard reflects the increasing digestive or metabolic capacity of birds. Kidney weight was almost double the lowest level of dNa in the case of NaHCO_3_ when compared with the lowest level of Na_2_SO_4_ (p ≤ 0.03; interaction effect). This suggests the bicarbonate buffer system mainly determines blood acid–base balance for optimal production performance and functions under regulatory control of the kidneys [14]. Kidney weight is indicates broiler dietary nutrient insufficiencies [34] or the presence of anti-nutritional factors [35, 36]. In the present study, the acid base imbalance might cause the higher kidney weight. A linear increase in dNa decreased spleen weight (p ≤ 0.02). The interaction (source × level) effect influenced the bursa weight (p ≤ 0.001). The low weight of the spleen was observed at 0.35% dNa. The low weight of the bursa was observed at 0.26% dNa for both salt sources. As the spleen and bursa are associated with immune function (as lymphoid organs) this may explain the poor DP at these levels.

## Conclusions

Birds showed increased breast and thigh meat yield, and increasing capacity of the gizzard at higher levels of dietary sodium. In contrast, a reduced dressing percentage with increasing supplementation of dietary sodium was unclear. Lower levels of dietary sodium were sufficient for supporting immune organs (bursa and spleen). Therefore a verification of requirements by changing other electrolytes (K^+^ and Cl^−^), keeping a constant DEB level, and changing salt sources is suggested.
